# Environment-dependence of behavioural consistency in adult male European green lizards (*Lacerta viridis*)

**DOI:** 10.1371/journal.pone.0187657

**Published:** 2017-11-07

**Authors:** Gergely Horváth, Boglárka Mészáros, Tamás János Urszán, Katalin Bajer, Orsolya Molnár, László Zsolt Garamszegi, Gábor Herczeg

**Affiliations:** 1 Behavioural Ecology Group, Department of Systematic Zoology and Ecology, Eötvös Loránd University, Budapest, Hungary; 2 Laboratório de Biogeografia e Macroecologia, Universidade Federal do Rio Grande do Norte Centro de Biociências, Departamento de Botânica, Ecologia e Zoologia, Campus Universitário, Lagoa Nova, Natal, Rio Grande do Norte, Brazil; 3 Department of Evolutionary Ecology, Estación Biológica de Donaña-CSIC, Seville, Spain; University of Pretoria, SOUTH AFRICA

## Abstract

Understanding the background mechanisms affecting the emergence and maintenance of consistent between-individual variation within population in single (animal personality) or across multiple (behavioural syndrome) behaviours has key importance. State-dependence theory suggests that behaviour is ‘anchored’ to individual state (e.g. body condition, gender, age) and behavioural consistency emerges through behavioural-state feedbacks. A number of relevant state variables are labile (e.g. body condition, physiological performance) and expected to be affected by short-term environmental change. Yet, whether short-term environmental shifts affect behavioural consistency during adulthood remains questionable. Here, by employing a full-factorial laboratory experiment, we explored if quantity of food (low *vs*. high) and time available for thermoregulation (3h *vs*. 10h per day) had an effect on activity and risk-taking of reproductive adult male European green lizards (*Lacerta viridis*). We focussed on different components of behavioural variation: (i) strength of behavioural consistency (repeatability for animal personality; between-individual correlation for behavioural syndrome), (ii) behavioural type (individual mean behaviour) and (iii) behavioural predictability (within-individual behavioural variation). Activity was repeatable in all treatments. Risk-taking was repeatable only in the low basking treatments. We found significant between-individual correlation only in the low food × long basking time group. The treatments did not affect behavioural type, but affected behavioural predictability. Activity predictability was higher in the short basking treatment, where it also decreased with size (≈ age). Risk-taking predictability in the short basking treatment increased with size under food limitation, but decreased when food supply was high. We conclude that short-term environmental change can alter various components of behavioural consistency. The effect could be detected in the presence/absence patterns of animal personality and behavioural syndrome and the level of individual behavioural predictability, but not in behavioural type.

## Introduction

Behaviour is known as one of the most plastic traits of animals, and classically viewed to allow immediate optimalisation upon environmental change [1–3]. Hence, between-individual behavioural differences were classically seen as non-adaptive variation around the adaptive mean of the population [4]. However, the statistical and biological significance of consistent between-individual behavioural variation (behavioural consistency) in single (animal personality) and across multiple (behavioural syndromes) behaviours became widely accepted recently [2,4–7]. There are several alternative but non-exclusive explanations for these patterns, with state-dependence being one [8–11]. From a behavioural ecology aspect, state is defined as all characters of an individual that alters the costs and benefits of behavioural decisions [11,12]. Inherently stable state variables (e.g. gender) can obviously result in stable between-individual differences in behaviour, but a large body of recent evidence proves that environment-dependent labile state variables (e.g. energy reserves, hormone levels) may create consistent behavioural variation too [9,13–16]. Several authors claim that short-term environmental fluctuations experienced early in life has the potential to alter behavioural consistency [17–26], yet, another question is whether environmental change could affect behavioural consistency during adulthood [11,27].

Among environmental factors, food (energy) availability is expected to drive behavioural strategies because (i) behaviour itself has direct energetic costs, (ii) different behaviours differently affect the probability of acquiring energy and (iii) different levels of energy reserves might translate to different optimal life-history strategies (e.g. growth *vs*. reproduction, current reproduction *vs*. future reproduction) directly linked to behavioural strategies [15,16,28,29]. Alongside food availability, body temperature is also highly important for ectotherms, because by affecting the speed of biochemical reactions, it ultimately affects individual performance from locomotion to capturing, handling and digesting prey [30–33]. Small-bodied reptiles control their body temperature almost entirely by means of behaviour, depending strongly on the thermal environment [31,34–37]. However, it is not yet studied how thermoregulatory possibilities are linked to behavioural consistency in behaviourally thermoregulating ectotherms.

Here, we aimed to study how availability of food and basking opportunities influence the behavioural consistency of reproductive adult male European green lizards (*Lacerta viridis*). Mating season of *L*. *viridis* is synchronized [38], hence, short-term differences in environmental conditions and individual state are expected to affect individual fitness, and eventually, behaviour. To answer this question, we ran a full factorial experiment with two levels of food (high *vs*. low) and basking treatments (behavioural thermoregulation possible for 3h *vs*. 10 h daily), manipulating available energy and individual physiological performance parallel. We assessed activity and risk-taking three times (on consecutive days) for every individual. First, we tested whether the treatments affected behavioural consistency *per se*, i.e. whether the presence/absence/strength of animal personality (repeatability of activity and risk-taking) and behavioural syndrome (between-individual correlation between activity and risk-taking) varied between the treatment groups. Second, we tested whether our treatments affected behavioural type (individual mean behaviour). Third, we tested whether the treatments affected behavioural predictability (within-individual variation unrelated to environmental change; see [39–42]). We note here that our assays testing behavioural consistency during a short time period do not test for animal personality in a classical sense. However, rapid assays, combined with a relatively long acclimation period (see below), may provide sufficient estimates of repeatability [43]. Moreover, considering the ecological context (short and synchronized mating season), we believe that our repeatability estimates are biologically informative and valid indicators of personality in this particular context. We hypothesized that since environmental variation is higher between than within treatments, between-individual divergence (strength of behavioural consistency) will be higher in the full sample than within treatment groups. We also hypothesized that behavioural consistency will be stronger under favourable (e.g. high food × long basking time treatment) conditions where extreme behavioural strategies are expected (see [27,44]). We should note here that an opposite pattern (i.e. stressful environments resulting in the expression of hidden between-individual variation) could be valid as well hypothetically, however, experimental data support the former [27,44]. We also expected variation in behavioural types, for instance, the asset protection theory predicts risk-averse behavioural strategy when individual assets are high (e.g. high energy reserves) [45].We are not aware of any general theories regarding environmental variation and behavioural predictability, besides the idea that high predation risk would lower behavioural predictability [40,41], hence, we had no clear expectations in this regard.

## Materials and methods

### Study animals

We noosed 38 adult male lizards during the mating season of 2014 (between 6 and 9 of May) near Tápiószentmárton, Hungary (47°20′25″ N, 19°47′11″ E). Lizards were transported to the laboratory of the Department of Ethology of Eötvös Loránd University and housed individually in opaque plastic boxes (80 cm × 40 cm × 40 cm, length, width, height, respectively). We provided peat as substrate and small black plastic boxes (15 cm × 8 cm × 7 cm, length, width, height, respectively) as shelters. After housing the animals, we let them acclimate for 11 days with food (mealworms, *Tenebrio molitor*) and water provided *ad libitum*, and basking opportunities (see below) provided for 10 hours per day. As eight or ten individuals were caught per day, behavioural tests (see below) were shifted according to the time of capture to provide the same time schedule for every individual. During the acclimation period, we took blood samples (on average 30 μl) from the post-orbital sinus on the fourth day and ran phytohaemagglutinin (PHA) tests, between the eighth and tenth days, for other scientific reasons to be discussed in a sister paper (Mészáros et al. in review). After this, animals were allowed one day to recover. These approaches are widely used without known negative effects on animals’ health and behaviour (e.g. [46,47]), thus we are convinced that these standardised procedures did not affect our results. All applicable international, national, and/or institutional guidelines for the care and use of animals were followed; the experiment was approved by the Animal Welfare Committee of the Eötvös Loránd University (permit number: MÁB PEI/001/444-4/2013). The experiment was performed under the licence of the Middle-Danube-Valley Environmental, Nature and Water Inspectorate (permit number: 7223-6/2014).

### Treatments

Treatments started on the 12 days after the capture and lasted for 26 days. We repeated blood-sampling 26 and PHA tests 30–32 days after capture for the aforementioned reasons. Behavioural assays started 35 days after capture (23 days after the treatments started) and lasted for three days, during which the treatments continued.

Individuals were randomly assigned to the low and high food treatments. In the low food group, lizards were provided 1 g mealworm every second day at 8.00 am (UTC+02.00), while high food males were fed daily with 5 g mealworm, starting at the same time. Within each food treatment, we randomly assigned individuals to the short and long basking treatments, differing in the available time for thermoregulation provided by 40 W spot lamps (OSRAM, Augsburg, Germany). Average substrate temperature beneath the spot lamps was 36.75°C (± 4.32°C [Standard Deviation; SD]). Lizards in the long basking group had 10 hours for thermoregulation from 7.00 am to 17.00 pm (UTC+02.00), while animals in the short basking group were provided only 3 hours for thermoregulation from 7.00 am to 10.00 am (UTC+02.00). As room temperature was set constantly to 18°C (mean ± SD = 18.14°C ± 0.46°C), lizards could not reach their preferred temperature range [48] when the heating lamps were off. The photoperiod (14 L:10 D) was provided by Repti Glo 2.0 Full Spectrum Terrarium Lamps (Exo Terra, Rolf V. Hagen Inc., Holm, Germany) which does not emit considerable heat. No lizard died, got injured or autotomized its tail as a result of handling, sampling or treatments. We chose these treatments because in an earlier study, similar treatments were successful in affecting body condition and the development of a sexually selected nuptial colour signal in the same population [38]. Thirty-eight individuals were used in the tests (sample sizes for the different treatment combinations: low food / short basking = 10; low food / long basking = 9; high food / short basking = 10; high food / long basking = 9). After the experiment we released all individuals at the capture site. We measured the body weight (BW) of the animals’ right before the start and at the end of the treatments to the nearest 0.1 g using a digital scale.

In a repeated measures General Linear Model (GLM), we found significant BW change among the individuals (repeated measures factor [repeat]: *F*_*1*,*34*_ = 8.03, *P* = 0.008) and according to our expectations, food treatment affected the BW change (food × repeat interaction: *F*_*1*,*34*_ = 5.92, *P* = 0.02; temperature × repeat interaction: *F*_*1*,*34*_ = 0.24, *P* = 0.63; food × temperature × repeat interaction: *F*_*1*,*34*_ = 0.36, *P* = 0.55). Lizards in the low food treatment maintained their original weight, while lizards in the high food treatment significantly increased it (supplementary material: S1 Fig). Therefore, we could not provide clear ‘stressful’ *vs*. ‘rich’ environments in terms of food, rather ‘normal’ *vs*. ‘rich’ environments.

### Behavioural assays

Behavioural assays took place between June 10–15, lasting three days for every individual, the exact time depending on the day of capture. Treatments before assays lasted for 23 days. We assume that we left sufficient time for the treatments’ effects to accumulate. The treatments also continued during the behavioural assays. Activity and risk-taking of individuals were tested three times in different days in the animals’ home boxes.

Activity was recorded between 7.30 am and 8.00 am (UTC+02.00) using web-cams (Microsoft LifeCam HD-3000, Microsoft Corp., Redmond, WA, USA). The video footages (30 min) was analysed using the MotionMeerkat program [49] to evaluate the time (sec) the animals spent moving. Risk-taking was measured on the same day, right after the activity assays between 9.00 am and 9.30 am (UTC+02.00). We performed risk-taking assays in the home boxes, as this method resembles animals’ reaction under natural circumstances more than it would in novel test arenas [50]. First, the experimenter (BM) caught the individual and placed it into its shelter box, then closed it with a removable cardboard door. This was done to mimic a situation when the lizard was caught by a predator, but managed to escape into its familiar refuge. Individuals were left in the box for 5 min. After this, the cardboard door was removed and the lizard’s behaviour was recorded using web-cams. Video recording was stopped 30 min after the experimenter finished the procedure with the last individual. Time till the lizards midbody (from head to cloaca) emerged from the shelter was used to evaluate risk-taking. The test order of the animals was randomized in every assay. Out of the 114 assays, individuals did not emerge during the 30 min observation period in only 2 cases. These observations received maximal score (1800 sec).

### Statistical analysis

Risk-taking was log_10_- and activity was square-root-transformed to achieve normal distributions of the model residuals. To estimate repeatability (i.e. animal personality) of activity and risk-taking in the pooled sample and the different treatment groups, we ran linear mixed models (LMM) using restricted maximum likelihood estimation in the lme4 package [51] separately, with the behavioural variable of interest as the dependent variable and individual as the random factor. Confidence intervals were calculated by nonparametric bootstrapping, while significance is provided by random permutation, both sampled at each 1000th iteration.

To test for between individual behavioural correlations (i.e. behavioural syndromes), bivariate mixed models (BMM) were used to partition variance and covariance components at different levels [52]. The models were fitted for the pooled sample and the different treatment groups separately, with the two behaviours as bivariate response variables and ‘individual’ as random factor, using the MCMCglmm R package [53]. This package implements a Bayesian framework for model fitting with long iterations (1.300.000 with 300.000 burn-in periods), the Markov chain was sampled at each 1000th iteration. Based on our BMM, we decomposed phenotypic correlations into between-individual and within-individual correlations, using the former as indicator of behavioural syndromes [7,52,54]. The results are given as correlation coefficients and their 95% credibility intervals.

To test whether the treatments affected mean behaviour, we ran LMMs on the behaviours separately. Activity and risk-taking were our response variables, snout-to-vent length (SVL; to the nearest 0.01 mm) treatments and their interactions were fixed effects and individual was a random factor. We added *z*-transformed order of trials both as single fixed effect and random slope (i.e. in interaction with individual) to the models to test for habituation on the group- and individual-levels directly.

GLMs were used to test for treatment effects on behavioural predictability. The SD of activity and risk-taking (as proxies for predictability) were the response variables and SVL, treatments and their interactions were added as fixed effects. Note that we applied a long acclimation period before assays, there were no sign of habituation (see Results) and the environment was standardised within treatments, hence, SD is a good proxy for behavioural predictability in our case. We applied backward stepwise model selection to remove nonsignificant effects from our LMMs based on the ‘step’ function provided by the package lmerTest [55], while a similar procedure was based on likelihood ratio test for the GLMs. We report marginal and conditional *R*^2^ for our models using the MuMIn R package [56]. All analyses were running in the R statistical environment [57].

## Results

### Animal personality and behavioural syndrome

Considering the relatively low sample sizes, we accepted animal personality being present when random permutations yielded significant results and the bootstrapping provided 95% confidence intervals excluding zero. Both activity and risk-taking were repeatable in the pooled sample (Table 1). Repeatability estimates revealed that activity had high repeatability in each treatment group (Table 1). Regarding risk-taking, we detected high repeatabilities in the short basking treatment irrespective of food availability (Table 1). Confidence intervals for the repeatability estimates between the groups and the pooled sample highly overlapped in all behavioural variables (Table 1).

**Table 1:** Repeatability estimates for activity and risk-taking of adult male *Lacerta viridis* in the pooled sample (All) and in the different treatment groups (HF = high food treatment, LF = low food treatment, LB = long basking time treatment, SB = short basking time treatment).

Behaviour	All (*N* = 38)	HF/LB (*N* = 9)	LF/LB (*N* = 9)	HF/SB (*N* = 10)	LF/SB (*N* = 10)
Activity	**R = 0.7**	**R = 0.53**	**R = 0.79**	**R = 0.66**	**R = 0.84**
	*P <* 0.001	*P* = 0.005	*P* < 0.001	*P* < 0.001	*P* < 0.001
	CI = 0.53–0.81	CI = 0.01–0.82	CI = 0.38–0.93	CI = 0.25–0.86	CI = 0.52–0.94
Risk-taking	**R = 0.46**	R < 0.001	R = 0.37	**R = 0.59**	**R = 0.55**
	*P* < 0.001	*P* = 0.85	*P* = 0.03	*P* = 0.001	*P* = 0.003
	CI = 0.24–0.63	CI = 0–0.46	CI = 0–0.69	CI = 0.15–0.82	CI = 0.02–0.8

Estimates are based on Linear Mixed Models (LMMs). Repeatabilities (R) and 95% confidence intervals (CI) are shown. Significance (*P*) estimates are based on random permutations.

Significant repeatabilities are in bold font.

**Fig 1:**
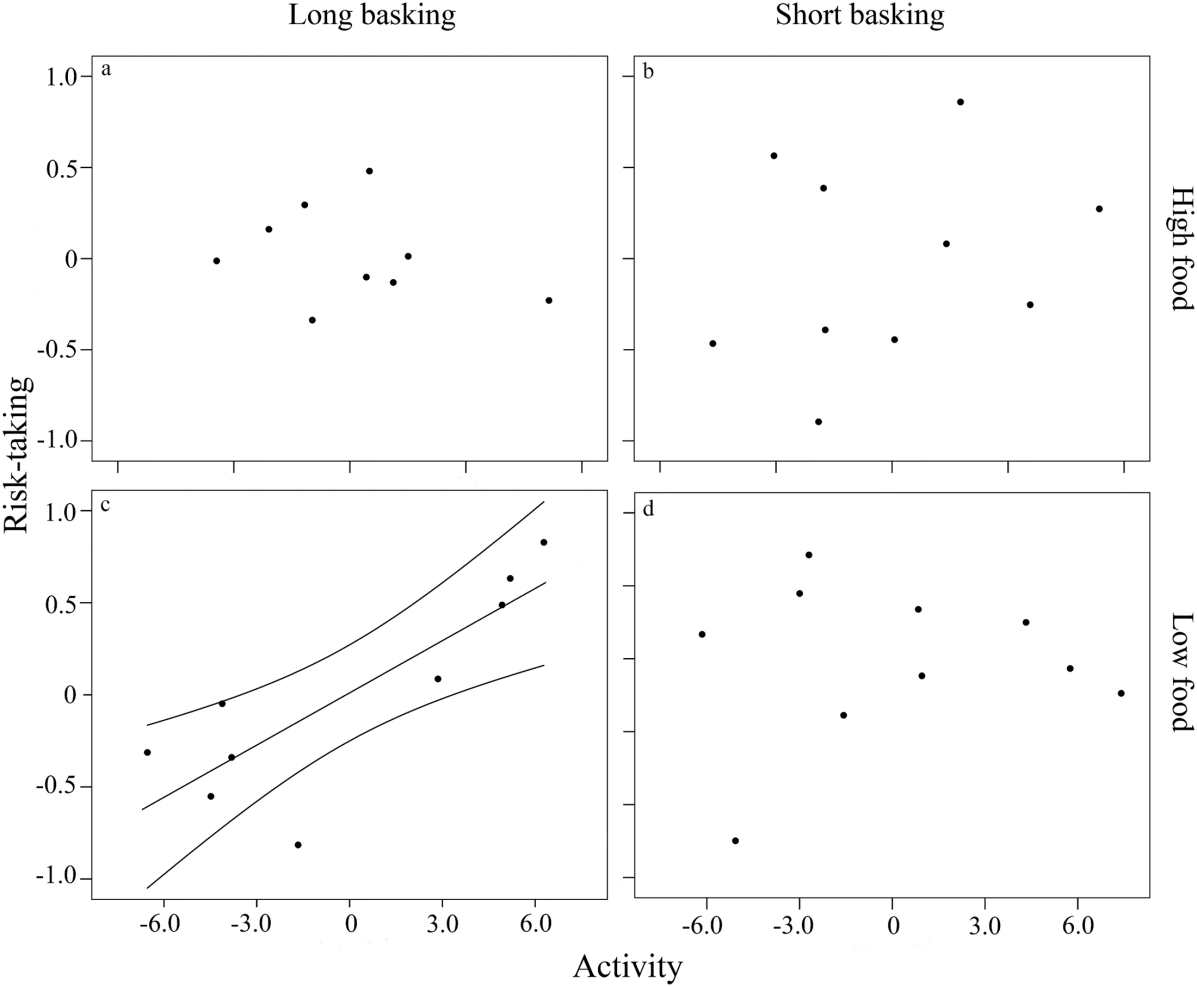
Between-individual correlation of risk-taking and activity in adult male European green lizards (*Lacerta viridis*) in the a) high food × long basking time (nonsignificant), b) high food × short basking time (nonsignificant), c) low food × long basking time (significant), and d) low food × short basking time (nonsignificant) groups. Risk-taking and activity are represented by best linear unbiased predictors (BLUPs) extracted from bivariate mixed models. Risk-taking is represented by a latency variable that was multiplied by -1 for straightforward interpretation; hence, small values translate to low risk-taking in the figure. Regression line is shown only for significant relationships.

Bivariate mixed models revealed nonzero between-individual behavioural correlation (i.e. behavioural syndrome) only in the low food × long basking time treatment group (Table 2). Here, we found a strong negative correlation between movement activity and latency to emerge from refuge after simulated attack (*r* = -0.74, 95% CI range = -0.94 –-0.003, Fig 1). Since risk-taking is estimated with a latency variable, this pattern translates to a positive correlation between the two behavioural traits. In the other treatment groups, between-individual correlations did not differ significantly from zero (Table 2). We note that our sample size was somewhat low for detecting weak correlations with high statistical power, but the pattern is clear regarding an existing treatment effect on the strength of the correlation.

**Table 2:** Between- and within-individual correlation between activity and risk-taking of adult male *Lacerta viridis* in the pooled sample (All) and in the different treatment groups (HF = high food treatment, LF = low food treatment, LB = long basking time treatment, SB = short basking time treatment).

	All (*N* = 38)	HF/LB (*N* = 9)	LF/LB (*N* = 9)	HF/SB (*N* = 10)	LF/SB (*N* = 10)
Between-individual	*r* = -0.29	*r* = 0.13	***r* = -0.74**	*r* = -0.25	*r* = 0.17
	CI = -0.58–0.14	CI = -0.67–0.87	**CI = -0.94 – -0.003**	CI = -0.81–0.52	CI = -0.79–0.58
Within-individual	*r* = -0.02	*r* = -0.27	*r* = -0.19	*r* = 0.24	*r* = -0.06
	CI = -0.26–0.21	CI = -0.49–0.26	CI = -0.71–0.21	CI = -0.07–0.73	CI = -0.33–0.48

Estimates are based on Bivariate mixed models (BMM) applying Markov Chain Monte Carlo (MCMC) approximation. Correlation coefficients (*r*) and 95% credibility intervals (CI) are shown.

Significant correlation coefficients are in bold font.

### Behavioural types and predictability

**Fig 2:**
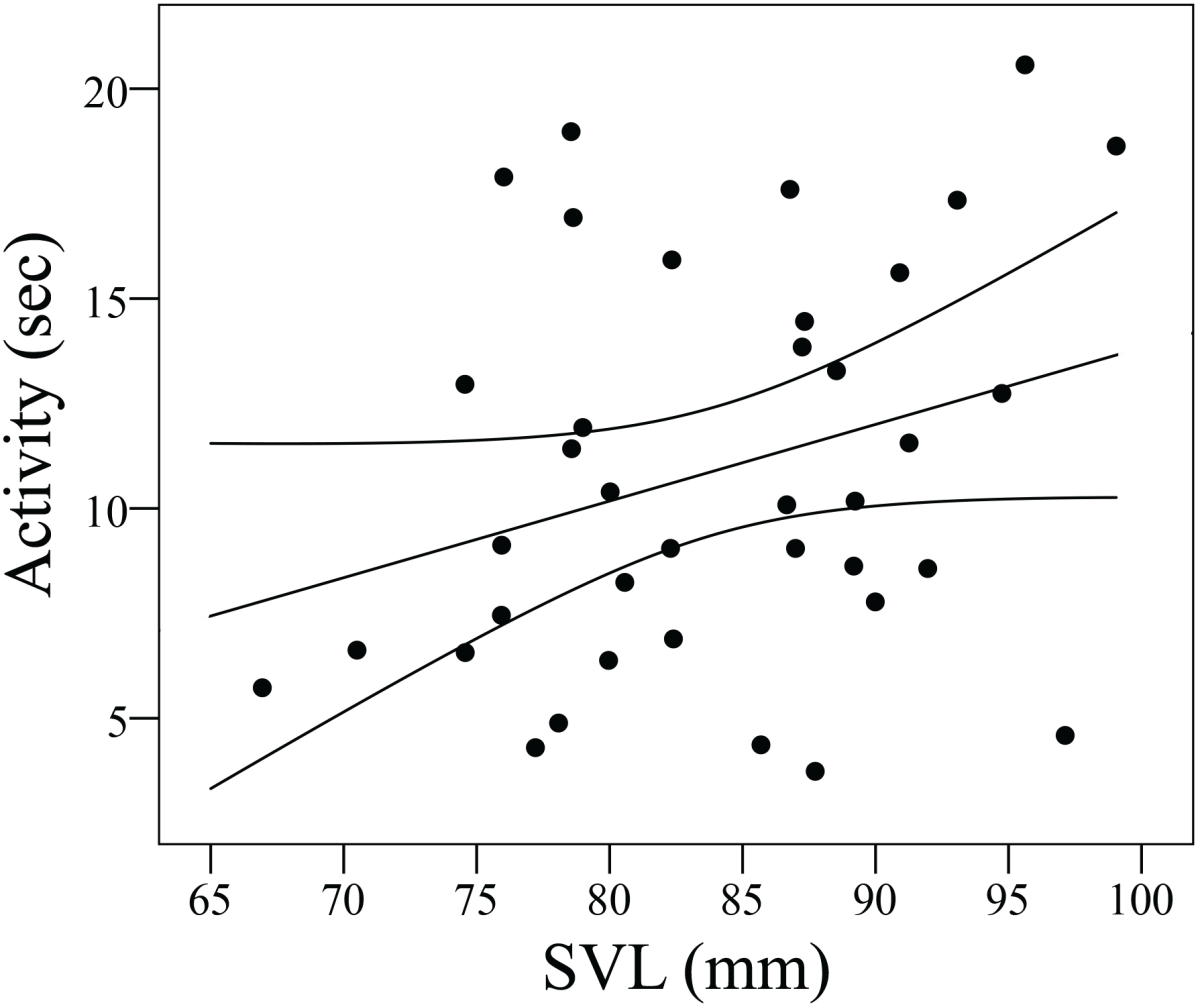
Significant relationship (*R*^*2*^ = 0.1) between activity and snout-to-vent length (SVL, mm) in adult male European green lizards (*Lacerta viridis*).

Our LMMs revealed a significant effect of SVL on activity, larger (≈ older) lizards being more active (*F*_*1*, *36*.*03*_ = 4.84, *P* = 0.034; Fig 2). Although we found no significant treatment effects on activity or risk-taking, there were marginally significant trends for larger individuals being more active in the long basking time treatment (*F*_*1*, *34*,*13*_ = 3.81; *P* = 0.06) and all lizards taking more risk in the short basking time treatment (*F*_*1*, *35*.*27*_ = 3.36; *P* = 0.08) (data not shown). We found no sign of habituation (activity: *F*_*1*, *69*.*41*_ = 0.58, *P* = 0.45; risk-taking: *F*_*1*, *68*.*86*_ = 1.82, *P* = 0.18) and individuals’ habituation trends did not differ from each other (activity: χ^2^ < 0.001, df = 1, *P* > 0.99; risk-taking: χ^2^ = 0.06, df = 1, *P* = 0.79). The fixed effects explained 19% (activity) and 18.5% (risk-taking) of the total variance, while the full models 69% (activity) and 46% (risk-taking), which can be seen as good explanatory power for behavioural variables. The remaining nonsignificant effects are reported in the supplementary material (S1 Table).

**Fig 3:**
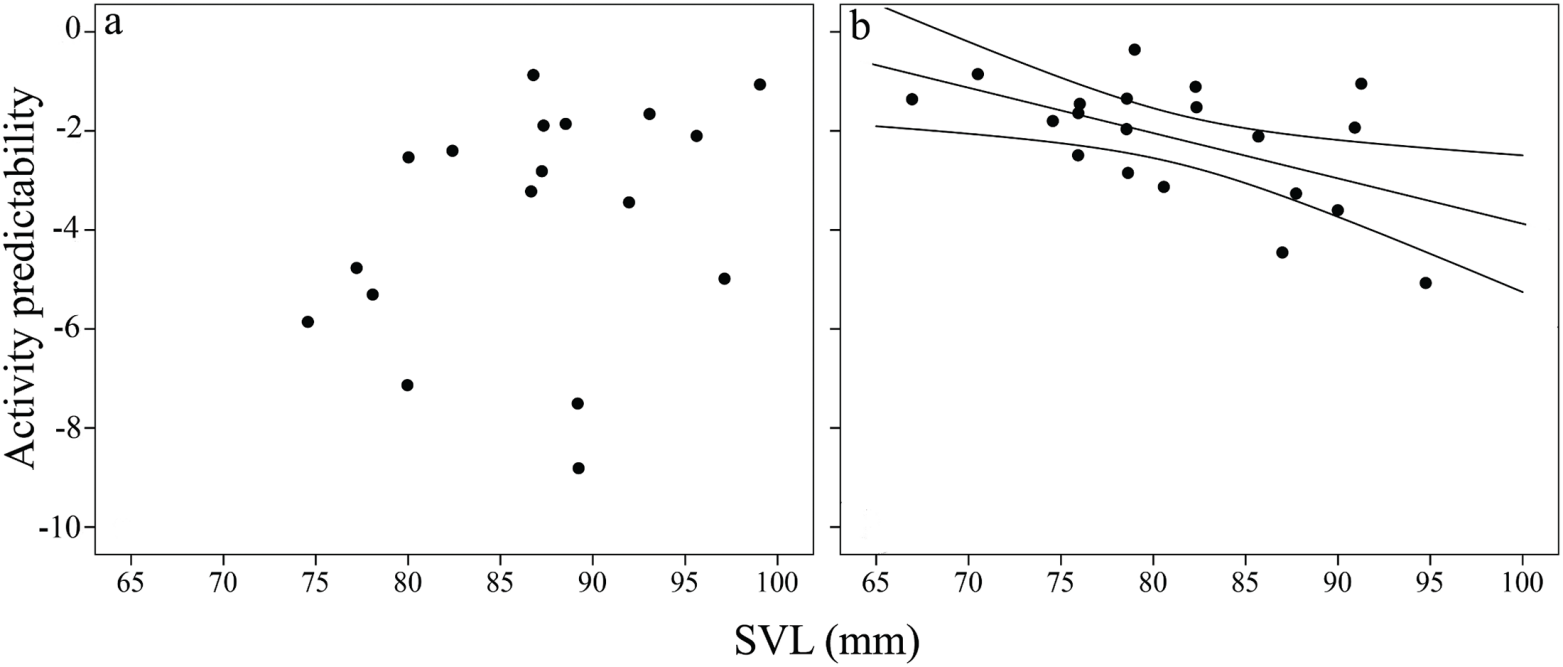
Relationship between predictability of activity (Standard Deviation) and snout-to-vent length (SVL, mm) in adult male European green lizards (*Lacerta viridis*) in the a) long basking time (nonsignificant) and b) short basking time groups (significant; *R*^*2*^ = 0.3). Predictability values were multiplied by -1 for straightforward interpretation; hence, small values translate to low predictability in the figure. Regression line is shown only for the significant relationship.

GLMs revealed a significant basking time effect on activity predictability (*χ*^*2*^ = 7.09, df = 1, *P* = 0.008), further, the SVL × basking time interaction was also significant (*χ*^*2*^ = 5.94, df = 1, *P* = 0.014). Lizards were less predictable in the long than in the short basking treatment (Least Squares means ± Standard Error [LSM ± SE]; long basking: 4.13 ± 0.48; short basking: 2.56 ± 0.45). To interpret the interaction, we ran two separate GLMs for the short- and long basking time treatments. We found a negative relationship between size and predictability in the short (*χ*^*2*^ = 7.73, df = 1, *P* = 0.005, Fig 3) but not in the long basking treatment (*χ*^*2*^ = 1.83, df = 1, *P* = 0.18, Fig 2).

**Fig 4:**
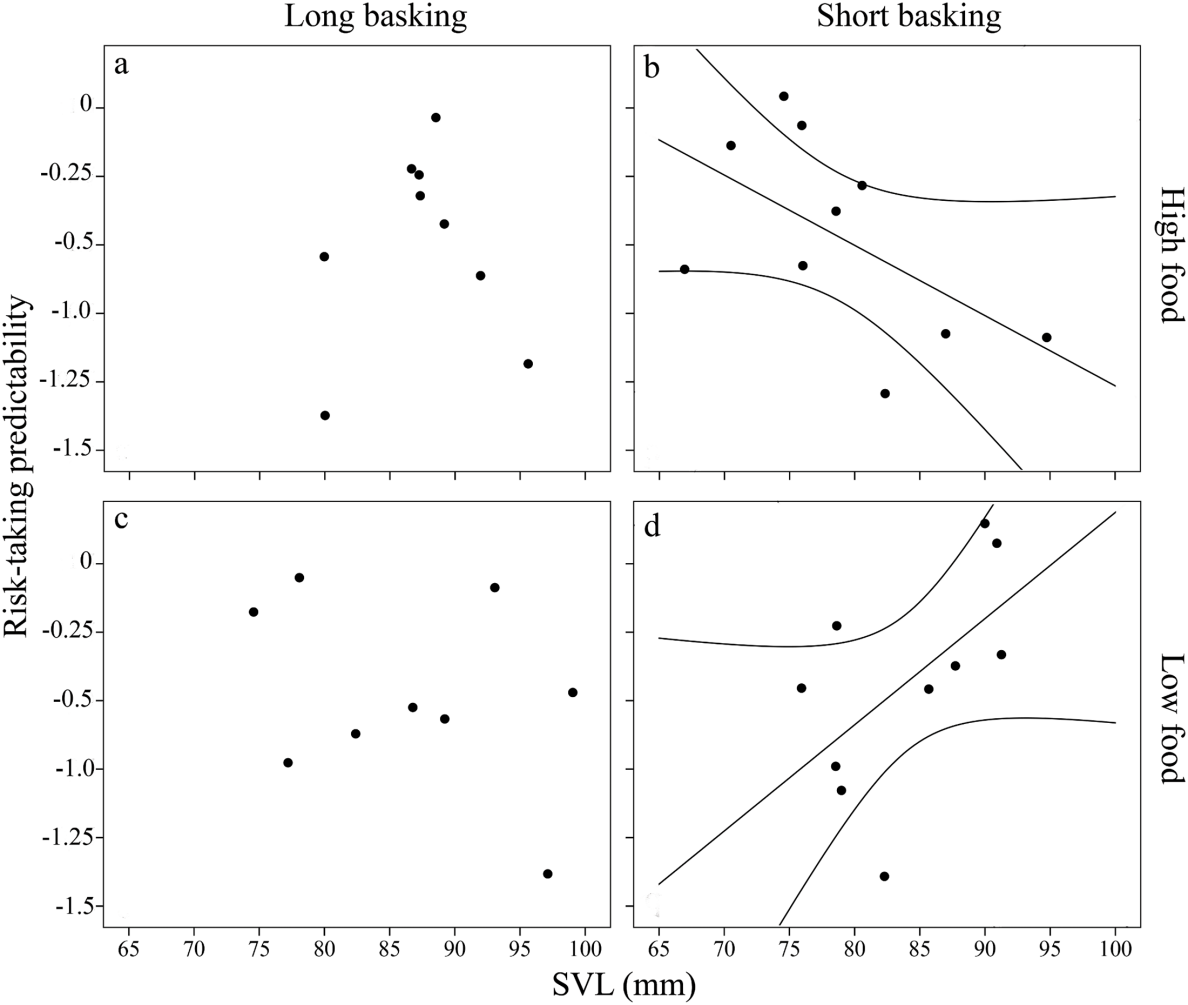
Relationship between predictability of risk-taking (Standard Deviation) and snout-to-vent length (SVL, mm) in adult male European green lizards (*Lacerta viridis*) in the a) high food × long basking time (nonsignificant), b) high food × short basking time (marginally significant; *R*^*2*^ = 0.32), c) low food × long basking time (nonsignificant), and d) low food × short basking time (significant; *R*^*2*^ = 0.33) groups. Predictability values were multiplied by -1 for straightforward interpretation; hence, small values translate to low predictability in the figure. Regression line is shown only for significant relationships.

Risk-taking predictability was affected by complex interactions. We found a significant food × basking time interaction (*χ*^*2*^ = 4.93, df = 1, *P* = 0.03). Within the long basking time treatment, food had no effect on predictability (LSM ± SE; high food: 0.66 ± 0.14; low food: 0.59 ± 0.12), however, high food treatment lizards were less predictable than low food treatment lizards in the short basking time treatment (LSM ± SE; high food: 0.75 ± 0.13; low food: 0.58 ± 0.11). Further, we found a significant SVL × food × basking time interaction (*χ*^*2*^ = 5.22, df = 1, *P* = 0.02). Here, we ran separate GLMs for the four treatment groups to reveal the nature of the interaction. Within the short basking treatment, we found a significant positive relationship between size and predictability in the low food treatment (χ^2^ = 3.88, df = 1, *P* = 0.048, Fig 4) and a marginally significant negative relationship in the high food treatment (χ^2^ = 3.74, df = 1, *P* = 0.053, Fig 4). In the high basking treatment, there were no size trends (all remaining nonsignificant effects are reported in the supplementary material [S2 Table]).

## Discussion

While several studies generally agree that the environment has crucial role triggering the emergence of animal personalities or behavioural syndromes during early ontogeny [17–20,24], evidence of environmental factors influencing the expression of behavioural consistency during adulthood has only recently started to accumulate, and the results are somewhat controversial [21–23,27,58–60]. Here, by manipulating available energy and time available for efficient physiological performance in reproductive adult male lizards, we found that these treatments indeed affected the strength of behavioural consistency both within and across the studied behaviours. While–quite surprisingly–the treatments did not affect behavioural types strongly, they did affect behavioural predictability and its relationship with size (an age proxy in lizards with indeterminate growth).

### Behavioural consistency

Our repeatability estimates indicate strong behavioural consistency for activity in the pooled sample and in all treatment groups compared to the average repeatability of behavioural traits, which was previously reported to be around 0.37 [61]. Further, confidence intervals of the treatment groups highly overlapped with that of the pooled sample, thus repeatability estimates between the treatment groups and the pooled sample did not differ. On the other hand, repeatability of risk-taking was moderate in the pooled sample, while repeatabilities were high in the short basking time treatment groups and low or negligible in the high basking time treatment groups. Therefore, we reject the hypothesis that lowered environmental variation (within treatment *vs*. pooled sample) should weaken animal personality. This is in line with our previous findings in another lacertid, the *Iberolacerta cyreni* [27]. Even though we found treatment effects on risk-taking consistency, the patterns reject our hypothesis about optimal circumstances resulting in ‘stronger’ personality due to the appearance of extreme behavioural strategies, which increase between-individual variation [44] and also contradict our previous results [27]. In *I*. *cyreni*, risk-taking consistency (estimated similarly to the present study) was missing only in the most challenging treatment, while consistency regarding shelter use was only present in the supposedly optimal treatment [27]. Here, risk-taking differed consistently between lizards only in the short basking time treatments, or with other words, in the treatments where daily physiological performance was suppressed by the thermally challenging environments. However, we note that during the risk-taking assays, thermoregulation was possible, hence, the increased between-individual variation cannot be a direct result of individual variation in risk-taking strategies during suboptimal locomotor performance. Rather, it reflects individual variation in decisions between safety *vs*. basking in environments where basking opportunities are generally limited, but actually available. Previous theoretical work from Luttbeg and Sih [9] suggests that stable between-individual differences emerges when resources and risk are matched (i.e. both are low, medium or high). It is a plausible explanation that when basking is limited, available basking spots represent an important resource, leading to the emergence of individual risk-taking *vs*. risk-averse strategies under high perceived predation risk. Or one can simply argue that–as opposed to Lichtenstein et al. [44]–it is not the optimal environment, but indeed some sort of challenge that triggers between-individual behavioural variation. At any rate, the contradicting results warrants further studies to establish the link between environmental challenge and strength of animal personality.

We found evidence for between-individual behavioural correlation only in one treatment (low food × long basking time). The detected syndrome can be seen as a particularly strong one (|*r*| = 0.74) compared to the mean estimate of phenotypic correlations in a meta-analysis (0.19; [7]. Therefore, we reject the hypotheses (i) that lowered environmental variation (within treatment *vs*. pooled sample) should weaken behavioural syndromes and also (ii) that optimal environmental conditions favour the emergence of extreme behavioural strategies strengthening the estimates of behavioural consistency. It seems that lowered environmental variation do not affect the strength of behavioural consistency during relatively short exposure (few weeks) in adult lacertid lizards ([27]; present study).

Regarding the biology behind the emerging syndrome in one of the treatment groups (or the collapsing ones in the others), we can only speculate. Our low food treatment did not result in detectable starvation, but it rather provided a control where individuals maintained their original weight. However, the increased physiological performance in the long basking treatment seems ubiquitous [31,62]. For instance, we found that basking time affected positively the nuptial colour development of male *L*. *viridis* in a previous study [38]. Therefore, we can say that behavioural syndrome was present only in the scenario where optimal physiological performance was coupled with average energy input. There is a possibility that challenging environments will induce behavioural strategies where seemingly independent behaviours (in our case, activity and risk-taking) will be linked and vary according to the given individual’s strategy [63]. Perhaps energy limitation coupled with high physiological potential in the short and intense mating period is somewhat stressful for male lizards. At any rate, our results support the notion that the presence/absence of behavioural syndromes can vary following environmental change in the same population. This was recently supported by a longitudinal study on the collared flycatcher (*Ficedula albicollis*), where not only the presence/absence, but even the direction of behavioural correlations varied across years as response to a varying environmental factor [64].

We note that our repeated behavioural measures were taken during three days, hence, our repeatability and between-individual correlation estimates cannot be used to prove or disprove animal personality and behavioural syndrome in the classical sense (i.e. consistent individual differences over longer periods of time). However, within our experimental framework, they are usable to test whether consistent between-individual differences were present in single or across multiple behaviours after more than three weeks of exposure to the different treatments. Hence, while our results are obviously inadequate to address questions about personality and syndromes during adulthood or even in the given year, they are indeed relevant indicators of personality and syndromes in the studied ecological context, the short and highly synchronised mating season.

### Behavioural type and predictability

We manipulated two environmental factors that are expected to have a profound effect on adult males’ fitness during the short mating season right after hibernation: available energy and time available for high physiological performance (needed for effective use of the gathered energy). We expected that the shortage of either food or basking time will make lizards bolder to exploit the limited utilities to the maximum. We note that the food treatment did affect body weight changes and in a previous study we showed that a similar basking time treatment had significant effect on the development of a sexually selected nuptial trait in the same population [38], hence, our treatments are effective. However, our treatments had only marginally significant effects on behavioural types: (i) within the long basking time treatment, larger animals were more active than small ones and (ii) lizards in the short basking time treatment took more risk than in the long basking time treatment. Larger lizards showing higher behavioural activity is in line with our previous findings [65]. However, it was only seen in the group where high physiological performance was available for 10 hours. Perhaps older males can and / or should risk more during the reproductive season if their physiological performance is high due to their superiority in male-male combats and lower future reproductive success [13,45]. The importance of maximum physiological performance during the mating season for males is ubiquitous, and since optimal body temperature in thermally challenging environments is reached *via* basking in small heliotherm lizards [34,66], increased risk-taking when basking time is limited can be expected. However, considering the marginal significance coupled with the low explanatory power indicated by the low marginal *R*^2^ estimates, the biological significance of these trends remains questionable.

It is noteworthy that since the experimental treatments were standardised, nonrandom variation in measurement error or environmentally induced plasticity can be ruled out as sources of systematic differences in within-individual behavioural variation. Therefore, as we did not find habituation, within-individual behavioural variation can be seen as describing behavioural predictability (*sensu* [39,42,67], i.e. an individual’s consistency in expressing its behavioural type in a given environmental situation). In contrast to the weak treatment effects on behavioural types, we found clear treatment effects on behavioural predictability. Lizards expressed their activity with lower predictability in the long than in the short basking time treatment. Further, we found that larger individuals’ activity was less predictable than their smaller conspecifics’, but only in the short basking time treatment. Risk-taking predictability was explained by complex interactions. Excess food decreased predictability in the short, but not in the long basking treatment. Further, we found size-dependence of risk-taking predictability, but only within the short basking time treatment, where food treatment changed the sign of the relationship: size had a negative effect in the high, but positive in the low food treatment. It is interesting that environmental effects on risk-taking predictability emerged only in the short basking time treatments, the same treatments where risk-taking consistency was significant. (see previous section). It suggests that thermoregulatory possibilities are important drivers of ectotherm behavioural strategies.

Again, we can only speculate regarding the exact biological mechanism in the background of these patterns. Recent experimental work on hermit crabs (*Pagurus bernhardus* [41,68]) and Eurasian perch (*Perca fluviatilis*; [69]) revealed that animals were less predictable at higher water temperature, which is in line with our results regarding activity predictability. Such patterns might be explained based on the direct environmental temperature–body temperature–metabolic rate link in ectotherms, where increased metabolic rate can lead to increased predation risk [70], which in turn can decrease behavioural predictability [40,41]. However, this explanation cannot be used to explain the different treatment × size interactions. It seems that different ecological contexts affect younger *vs*. older male lizards’ behavioural strategy, or more precisely, the rigidity of the individual behavioural type, differently. Attempting to explain these patterns would be premature at this stage. The accumulating evidence for environment-induced variation in behavioural predictability clearly indicates that it might depict a biologically relevant aspect of between-individual behavioural variation, in addition to behavioural type (mean behaviour) and behavioural plasticity (environmentally induced change in mean behaviour) and can vary independently from these other attributes [67,71].

### Conclusions

Taken together, we reported a case where experimentally induced short-term environmental variation affected the expression of behavioural consistency in adult animals both in presence/absence patterns of animal personality and behavioural syndromes, and in the variation of individual behavioural predictability. This, together with our previous results on *I*. *cyreni* [27] support the notion that–at least in lacertid lizards–consistent between-individual behavioural differences can be induced or dissolved by environmental factors during adulthood. Hence, the strength of natural selection operating on basic behavioural traits like movement activity or risk-taking could vary greatly depending on the environment, not only because the costs and benefits of expressing certain phenotypes depends on the environment, but also because between-individual variation is not constant. Further, our results strengthen the idea that behavioural predictability itself is a relevant individual trait, responding to ecologically relevant environmental stimuli or correlating with individual attributes.

## Supporting information

S1 Fig Change in body weight of adult male European green lizards (*Lacerta viridis)* between the low- and high food treatment groups. Body weight change is represented by group specific means of individual slopes. 95% confidence intervals are shown.
(TIF)

S1 Table Results of LMMs on activity and risk taking. *F* statistics (numerator and denominator df in parentheses) and *P* values are shown. Significant effects are in bold font. SVL = snout to vent length; basking = basking time treatment; food = food treatment.
(DOCX)

S2 Table Results of GLMs on predictability of activity and risk taking in the pooled sample and in the different treatment groups. First, we ran separate GLMs on the pooled sample with all possible interactions for activity predictability and risk-taking predictability. Second, based on the highest order significant interaction for the given behaviour, we ran separate GLMs for the treatments involved in the given interaction. Likelihood ratio Chi-square test statistics and *P* values are shown for df = 1. Significant effects are in bold font. SVL = snout to vent length; basking = basking time treatment; food = food treatment.
(DOCX)

S3 Table Activity and risk-taking of the assessed adult male European green lizards (*Lacerta viridis*). Raw data are shown.
(XLSX)

S4 Table Snout to vent length (SVL) and body weight of the assessed adult male European green lizards (*Lacerta viridis*). (XLSX)
