# Supplementary figures and images for: Environment-dependence of behavioural consistency in adult male European green lizards (Lacerta viridis)
Source: PLoS One. 2017 Nov 7;12(11):e0187657. doi: 10.1371/journal.pone.0187657 (PMC5675404; doi:10.1371/journal.pone.0187657)

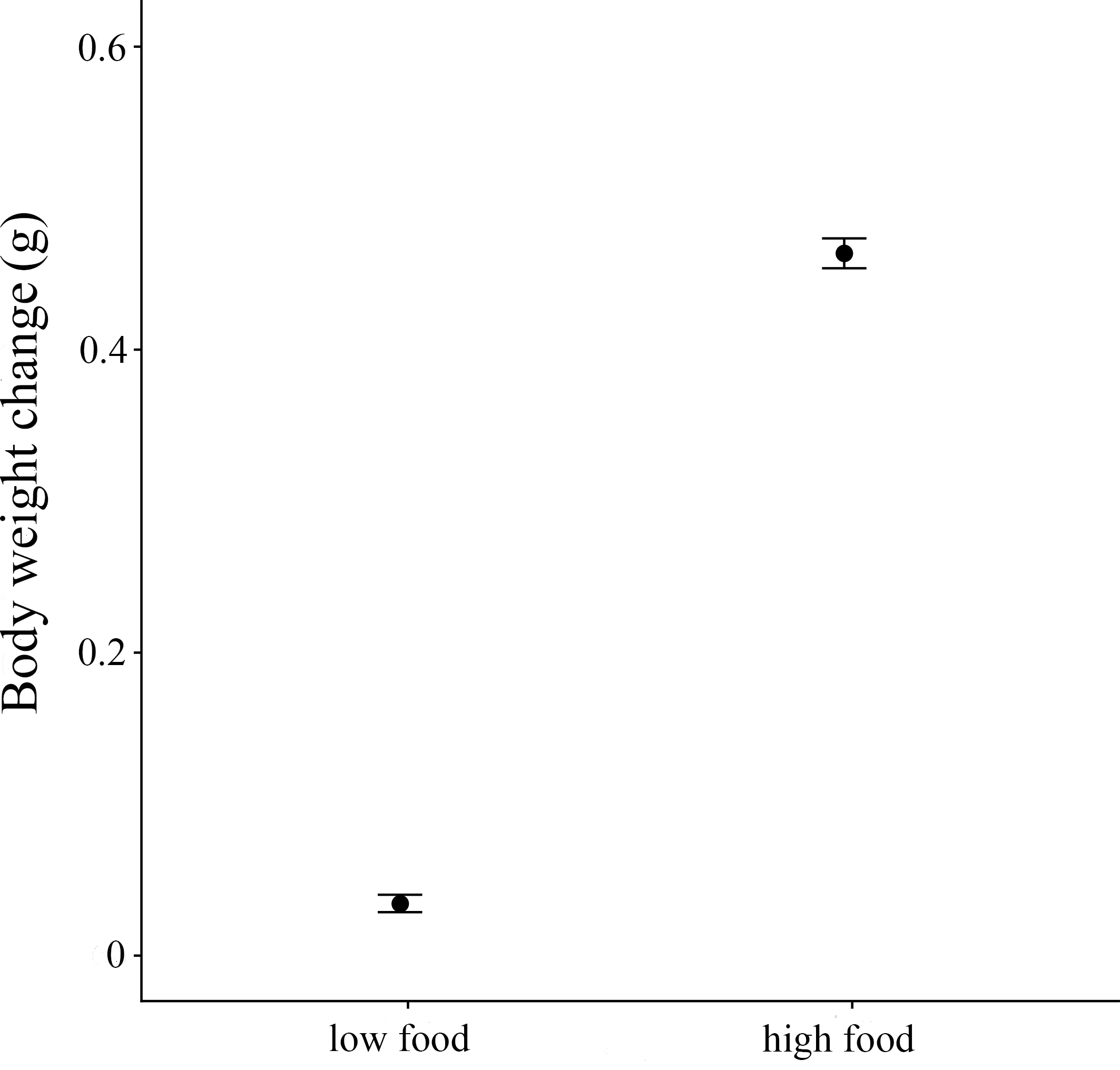

Supplement: S1 Fig — Body weight change is represented by group specific means of individual slopes. 95% confidence intervals are shown. (TIF) [file pone.0187657.s001.tif]
