# Supplementary material for: Environment-dependence of behavioural consistency in adult male European green lizards (Lacerta viridis)
Source: PLoS One. 2017 Nov 7;12(11):e0187657. doi: 10.1371/journal.pone.0187657 (PMC5675404; doi:10.1371/journal.pone.0187657)
Supplement: S1 Table — F statistics (numerator and denominator df in parentheses) and P values are shown. Significant effects are in bold font. SVL = snout to vent length; basking = basking time treatment; food = food treatment. (DOCX) [file pone.0187657.s002.docx]

| **Fixed effects** | **Activity** | **Risk-taking** |
| --- | --- | --- |
| SVL | **4.84 (1, 36.03); 0.034** | 1.51 (1, 36.11); 0.23 |
| basking | 0.014 (1, 35.19); 0.91 | 3.36 (1, 35.27); 0.08 |
| food | 0.03 (1, 32.74); 0.86 | 0.2 (1, 33.7); 0.66 |
| basking × food | 0.19 (1, 30.87); 0.66 | 2.01 (1, 32.72); 0.17 |
| basking × SVL | 3.81 (1, 34,13); 0.06 | 2.76 (1, 33.56); 0.11 |
| food × SVL | 1.46 (1, 31.57); 0.24 | 0.8 (1, 32.11); 0.38 |
| basking × food × SVL | 0.18 (1, 29.63); 0.68 | 0.18 (1, 31.50); 0.68 |
| repeat | 0.58 (1, 30.87); 0.45 | 1.81 (1, 68.86); 0.18 |
