# Supplementary material for: Environment-dependence of behavioural consistency in adult male European green lizards (Lacerta viridis)
Source: PLoS One. 2017 Nov 7;12(11):e0187657. doi: 10.1371/journal.pone.0187657 (PMC5675404; doi:10.1371/journal.pone.0187657)
Supplement: S2 Table — First, we ran separate GLMs on the pooled sample with all possible interactions for activity predictability and risk-taking predictability. Second, based on the highest order significant interaction for the given behaviour, we ran separate GLMs for the treatments involved in the given interaction. Likelihood ratio Chi-square test statistics and P values are shown for df = 1. Significant effects are in bold font. SVL = snout to vent length; basking = basking time treatment; food = food treatment. (DOCX) [file pone.0187657.s003.docx]

|  | **Fixed effects** | **Activity predictability**  **(*χ^2^*; *P*)** | **Risk-taking predictability**  **(*χ^2^*; *P*)** |
| --- | --- | --- | --- |
| **Pooled sample** |  |  |  |
|  | SVL | 3.16; 0.08 | 0.06; 0.8 |
|  | basking | **7.09; 0.007** | 1.16; 0.28 |
|  | food | 0.18; 0.67 | 0.52; 0.47 |
|  | basking × food | 0.73; 0.39 | **4.93; 0.03** |
|  | basking × SVL | **5.94; 0.014** | 1.28; 0.26 |
|  | food × SVL | 0.39; 0.53 | 0.52; 0.47 |
|  | basking × food × SVL | 0.64; 0.42 | **5.22; 0.02** |
| **Treatment groups** |  |  |  |
| long basking  short basking | SVL | 1.84; 0.18 | -- |
|  | SVL | **7.73; 0.005** | -- |
| high food × long basking | SVL | **--** | 0.05; 0.82 |
| low food × long basking | SVL | **--** | 1.05; 0.31 |
| high food × short basking | SVL | **--** | 3.74; 0.053 |
| low food × short basking | SVL | **--** | **3.88; 0.048** |
